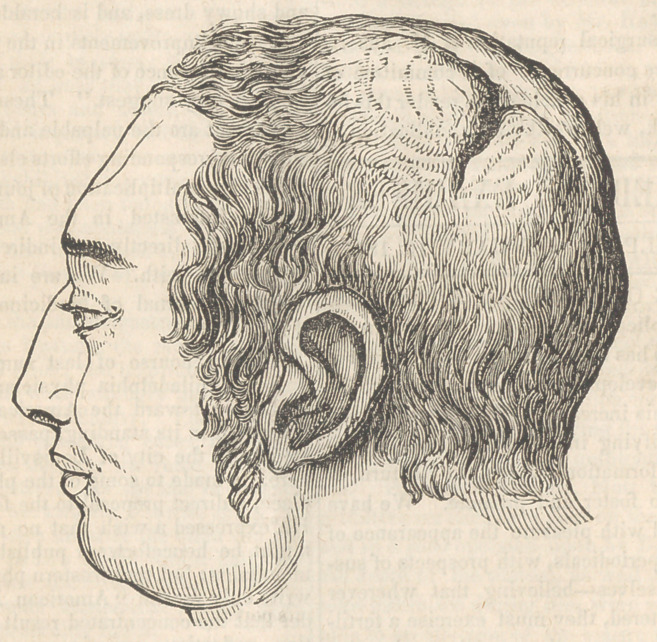# Domestic

**Published:** 1841-01-16

**Authors:** 


### DOMESTIC.

*Report of an Operation for the Removal of a Large Bony Tumour, called Spina Ventosa of the Skull.* By GEORGE M'CLELLAN, M. D., Professor of Surgery in the Pennsylvania Medical College.

In the early part of the month of December, 1838, Thomas Richardson, a resident of the city of Pittsburg, called on me for surgical aid. He was then twenty-two years of age, and had been afflicted about three years with a tumour upon the vertex of his skull. About six months before the first appearance of the tumour, he received a severe blow from a missile on the affected region, after which he occasionally experienced tenderness and pain there. As the tumour gradually increased, it produced a determination of blood to the head, attended with a sense of fulness and giddiness on stooping. But he was not deprived of any intellectual power; nor were any of his sensations or muscular actions disturbed.

The tumour was very hard and unyielding, and had been pronounced to be an *exostosis* by every surgeon who had examined it. It was oblong in shape, being four inches in the long, and three and one-fourth in the short diameter. It was raised in the centre about one and three-fourth inches above the surrounding portions of the outer table of the skull, and extended from about an inch beyond the sagittal suture on the right side obliquely to the left, and backwards over the adjacent portion of the left parietal bone. It occupied, phrenologically, the organs of Firmness, Self-esteem, Approbativeness, and a part of Cautiousness, on the left side.

I was induced to undertake an operation for extirpating this tumour, chiefly because no symptoms of cerebral affection could be discovered other than those which a moderate determination of blood to the head might produce. Two long incisions were first made at right angles near the centre of the swelling, and afterwards the scalp was dissected up from the whole surface, and, to some extent, around the sound bones. With a long narrow saw, held in a tangent to that portion of the circumference of the cranium, I then cut off the entire tumour, apparently at its base. The saw moved with difficulty while it was passing through the external table, but with great ease when it was acting upon the interior of the mass. This first led to the suspicion that the disease was not an *exostosis;* and when the prominence had been removed, it was made evident that a far worse state of things had to be encountered. The exposed surface presented perpendicular cells, or cavities, like those of a honeycomb, which were filled with a bloody, or pulpy and sanious matter. The case was at once decided to be a *spina ventosa* of the skull, and it was therefore deemed necessary to extract the whole mass from the surface of the dura mater beneath. A long and tedious extension of the operation was then undertaken. The whole mass of the tumour was circumscribed by the circular edge of a small Hays's saw, and the mass was pried out in successive fragments by an elevator, occasionally aided by the bone nippers and forceps. This part of the operation proved exceedingly difficult, for the tumour extended inwards much deeper below the internal table, than its outer surface had risen above the external table of the skull. Finally, however, a removal of the whole morbid structure was effected, and the dura mater was exposed, thin and livid in appearance, at the bottom of a deep cavity which the bystanders estimated to be capable of holding four and one-half ounces of water. There were no pulsations visible, although the circulation was strong and full. Some small spiculæ of bone adhered to the dura mater, which were extracted by the aid of forceps. In extracting the last of these, which appeared to penetrate the dura mater, a prodigious gush of venous blood issued, after which the patient fell into a convulsive syncope. The hæmorrhage was supposed to proceed from the longitudinal sinus, and was therefore arrested by graduated compresses and a bandage. The angles of the wound were brought as near together as possible over the compresses, for the purpose of affording support to them while they were confined by the bandages. Very little irritation resulted from this operation.

In nine days the compresses were loosened by suppuration, and, on removing them, the whole of the exposed surface was found to be granulating, and the orifice in the great sinus was closed. But the brain had not risen up to occupy the exposed cavity; and it was found impossible to place the flaps of the scalp in contact with the dura mater in the usual way, so as to close the wound. Mild dressings of patent lint were applied over the surface, and confined with moderate pressure by means of a double-headed roller. On the twelfth day after the operation, the cavity below the bone was evidently diminished, and every day thereafter it continued to decrease, until, in the fourth week, the surface of the brain covered by the granulating dura mater had risen up to the level of the inner table. The natural pulsatory motions did not appear, however, until the cavity was nearly filled; and, in the mean time, forcible pressure could be made on the surface of the brain without exciting any degree of stupor or inconvenience on the part of the patient. But as soon as the pulsations began to appear, every kind of pressure proved irritating to the brain. At the same time, a remarkable change took place in the character and bearing of the patient. He then became exceedingly timid and irresolute. It would render him pale and almost pulseless to approach him with a pair of scissors for the purpose of trimming away his hair from the margins of the wound; and the sight of a piece of lunar caustic, or a pair of forceps, in the surgeon's hands, would throw him into great trepidation. This state of his mental faculties continued for a long period after his complete recovery from the wound. He could not even go down into a cellar containing some plaster busts, without a sense of faintness and sinking; and the operation of taking a cast of his head in plaster, nearly prostrated all the functions of his mind and body. His carriage also became remarkably affected. Instead of maintaining his natural erect posture and bearing, he sunk his head and shoulders into an awkward stoop, and looked timidly and anxiously forward, as if he was afraid of blundering against a door post.

At the time of the operation, and until the pulsations of the exposed portion of his brain returned, he was remarkable for his firmness of mind and resolution. No patient ever bore a severe and protracted operation with more intrepidity. He sat upright in a chair, without any confinement, until the blood-vessel gave way at the close of the operation; and during its performance, he repeatedly inquired of the bystanders if it was the brain which was coming out under the efforts of the surgeon. It has been, moreover, stated by those who have known him well for years, that previous to this injury he had always been distinguished for his firmness, courage, and independence.

He is now (two years after the operation) living in perfect health at the Exchange Hotel, Pittsburg. He is engaged in active business, and is entirely exempt from any symptom of a return of the disease. His former firmness and intrepidity of mind have been gradually returning for a year past, and at present no departure from a healthy condition of mind or body can be discovered. A thickening or induration of the flaps of the scalp, which resulted from their long exposure and separation from the subjacent dura mater, and which at one period gave origin to a report that the disease had reappeared, has become entirely softened down, and attenuated by the natural process of absorption.

As this case occurred during the period of Mr. Combe's first course of lectures in Philadelphia, it excited great attention among all phrenologists. One of the gentlemen who attended the operation, addressed a letter to Mr. Combe, stating that both organs of Firmness were lost or destroyed ; and asked for an explanation of the apparent contradiction in the conduct of the patient to the principles of phrenology. Mr. Combe read this letter publicly to his class, and endeavoured to explain away the difficulty, by locating the position of the tumour posteriorly to the organs of Firmness. On a further, and subsequent examination of the wound, however, he decided that a great portion of the skull, over the region of Firmness, had been removed, together with that of several of the neighbouring organs, as I have enumerated them.

In no respect, however, does this case militate against the principles of phrenology. The organs, instead of being destroyed, were merely displaced or depressed by the growth of the tumour, in the same way that deformities are produced in some of the savage tribes by gradual pressure of the skull. Perhaps a better analogy may be drawn between the state of these organs and the parts of the brain pressed upon by internal effusions of blood, and depressed fractures, which do not produce the symptoms of compression. A compensation is then made for the space occupied by the effused blood or depressed bone, by a corresponding amount excluded from the cavity of the vessels, and retained in the general circulation.

A careful examination of this case will, I think, elicit observations in support of phrenology. The tone and excitement of the depressed region of the brain must probably have been increased by the invasion of the tumour, on the same principle that the muscles of labouring men are sometimes supported by leathern straps and bandages. On the other hand, the extirpation of the tumour must have had the same effect in removing the tension and mechanical support of the organs, as tapping for abdominal dropsy exerts upon the viscera of that great cavity. As soon as the depressed convolutions began to be unfolded or distended by the pulsation of the blood-vessels, they experienced a want of that pressure which had before stimulated them into an increase of activity. Their tone then became enfeebled, and continued so until the scalp had contracted adhesions to the outer surface of the dura mater, and the cicatrix became consolidated, so as to afford a firm and counteracting support to the pressure of the circulation below.

While Mr. Richardson was recovering from the operation, he was visited by several phrenologists for the purpose of establishing the precise location of the wound. Although they differed in their opinions in regard to the degree in which the organ of firmness was involved, they all agreed that Self-esteem was affected, and some thought the injury extended also to the organ of Concentrativeness. Inquiries were therefore directed by them to the manifestations of these faculties; and the patient did suggest some points of character in relation to which he conceived he had undergone an alteration. He asserted that he had for a long time previous to the operation lost his self-respect in the presence of company, and his power of confining his mind to any particular train of thought. But these peculiarities were not obvious to me, or to any of his familiar friends; and I have not thought it right to put them down in my estimate of his condition, as affected by the operation. Such affections may have been the result of that confusion in the mind which generally accompanies excessive determination of blood to the head. It has been suggested that they were produced by a paralysis of those organs which were most severely depressed by the deepest portion of the tumour; while, at the same time, the convolutions which lay under the edges of the tumour, and were only slightly pressed upon by it, were stimulated into increased activity of their functions. I will leave the decision of this point, however, to more experienced phrenologists, trusting that the facts which I have here given, will be judged of according to their merits.

*Am. Phren. Journ.*

## Figures and Tables

**Figure f1:**